# mGem: Decoding transmicrobe messaging—the growing impact of extracellular vesicles

**DOI:** 10.1128/mbio.03130-24

**Published:** 2025-04-29

**Authors:** Alicia Rojas

**Affiliations:** 1 Laboratory of Helminthology, Faculty of Microbiology, University of Costa Rica, San José, Costa Rica; 2 Centro de Investigación en Enfermedades Tropicales, University of Costa Rica, San José, Costa Rica; Instituto Carlos Chagas, Curitiba, Brazil

**Keywords:** EVs, coinfection, bacteria, fungi, protozoa, helminth, arthropod, tropical disease

## Abstract

Extracellular vesicles (EVs) are membrane-enclosed nanoparticles that contain proteins, lipids, and nucleic acids, playing key roles in interactions between pathogens and hosts. Most research on pathogen-derived EVs has focused on understanding their impact on disease pathogenesis, immunomodulation, and their use as biomarkers for diagnosis. However, few studies have explored the cross talk between bacteria, fungi, protozoa, helminths, or arthropods via EVs. This is particularly relevant in the human gut microenvironment, where a high diversity of microbes exists and is modulated with helminth gastrointestinal infections. Additionally, during blood-borne coinfections like malaria and lymphatic filariasis, direct communication between pathogens may take place, and in the arthropod-pathogen interface, the multiplication of some protozoa or helminths is essential for their development. Understanding transmicrobe EV communication may reveal novel therapeutic strategies for controlling infectious diseases in both vertebrate and invertebrate hosts, particularly in regions with high coinfection rates.

## PERSPECTIVE

Extracellular vesicles (EVs) are membrane-enclosed nanoparticles containing proteins, lipids, and nucleic acids (1) with mRNA, miRNA, lcRNA, tRNA (1), and even active proteasome subunits (2). EVs may be classified according to their size or their mechanisms of origin, and over the years, this classification has become more intricate with the increased resolution of analytical tools, now allowing the description of exomeres as small as 30 nm (3). Virtually, every biological system or fluid releases or contains EVs, with examples found in unicellular bacteria (4–6), ticks (7–9), and helminths (10–12) or detected in tears (13–15), milk (16–18), and snake venoms (19–21).

## PATHOGEN-DERIVED EV BIOGENESIS

Most knowledge about EV composition, biogenesis, and uptake pathways has been gained from studies using mammalian models (22). For instance, the mechanisms involved in multivesicular body formation, fusion with the plasma membrane, intraluminal vesicle biogenesis, and microvesicle development have led to the identification of cellular markers that help scientists track EVs to different cellular compartments, elucidate biogenesis pathways, and use EVs as disease biomarkers (23). This information has been useful in the study of organisms from other kingdoms different from Animalia. For example, some components of the endosomal sorting complex required for transport (ESCRT) machinery, typically involved in exosome formation in mammalian models, may be absent from these pathogens’ genomes (24), so they have explored alternative pathways to release EVs. Such mechanisms include the exploitation of the host’s intracellular EV machinery or the use of different and undescribed pathways in protozoa (25), fungi (26), helminths (27), or arthropods (28).

EVs are produced in bacteria as outer membrane vesicles and released after undergoing various ultrastructural changes in the bacterial cell (4). In contrast, protozoa typically exploit their intracellular habitat and use their host’s cell machinery to release EVs, as seen with the malaria-causing agent *Plasmodium falciparum* (29) or the amastigotes of *Leishmania infantum* (30). EVs from fungi involve breaching a thick and impermeable cell wall, requiring the use of alternative mechanisms compared to those described in other unicellular pathogens (31). Furthermore, helminths, mosquitoes, and ticks exhibit advanced differentiation into organs, systems, and life stages resulting in highly diverse EVs (7, 32). The mechanisms by which EVs are produced in these pathogens remain elusive, and most knowledge has been inferred through the identification of the ESCRT components in the organism’s genome or in the EVs themselves (7, 24, 33).

## PATHOGEN-DERIVED EV ROLES

The roles of pathogen-derived EVs have primarily been focused on their interactions with mammalian host cells. To name just a few examples, it has been demonstrated that resistance genes and miRNAs can be transferred from *P. falciparum-*infected red blood cells (RBCs) to uninfected RBCs, monocytes, and macrophages via EVs, leading to changes in the host immune responses (29, 34, 35). Furthermore, *Trypanosoma brucei* EVs can lead to erythrocyte clearance and anemia (36). EVs associated with the human fungal pathogen *Candida albicans* can activate type I interferons as an early innate immune response (37) and use EVs to promote their own growth and survival in the host (38). Moreover, EVs derived from the liver fluke *Fasciola hepatica* can remodel the host’s extracellular matrix and reduce the proliferation of hepatic stellate cells with anti-inflammatory responses in hepatocytes (39).

The release of EVs in multicellular organisms has been studied with the heartworm *Dirofilaria immitis*. Different EV miRNA and protein content were observed among EVs isolated from adult and larval worms (40), suggesting potentially different interactions with their mosquito and vertebrate hosts. To complicate matters further, EVs may have different biochemical profiles when isolated from different parasitic tissues (10). Therefore, if we consider the study of multicellular parasite EVs as a package or focus on only one life stage, we may under- or overestimate the effects of EVs on mammalian cells. Therefore, studies using parasitic EVs should focus on the EVs derived from a single life stage or parasite organ when possible. Additionally, these pathogens are usually in the presence of other coinfecting agents, thus affecting EV release, composition, and effects.

## STUDYING TRANSMICROBE CROSS TALK VIA EVS

Coinfections often lead to negative consequences for human health due to the potential synergistic effects of pathogens on the host’s immune system (41). Strikingly, more than 80 countries are endemic to two or more neglected tropical pathogens, with four countries having six or more of these agents and more than 90% of the infection burden occurring in Africa (42). Furthermore, 14 countries have reported coinfections by malaria and lymphatic filariasis by *Wuchereria bancrofti* (LF), with a prevalence of 11% (43), and 13 African countries are co-endemic for malaria and soil-transmitted helminths (STH) (44). Therefore, studying transmicrobe communications not only addresses the reality faced by a large portion of the world’s population but may also uncover relevant insights into how immune responses were regulated before health conditions were improved in the 20th century.

**Fig 1:**
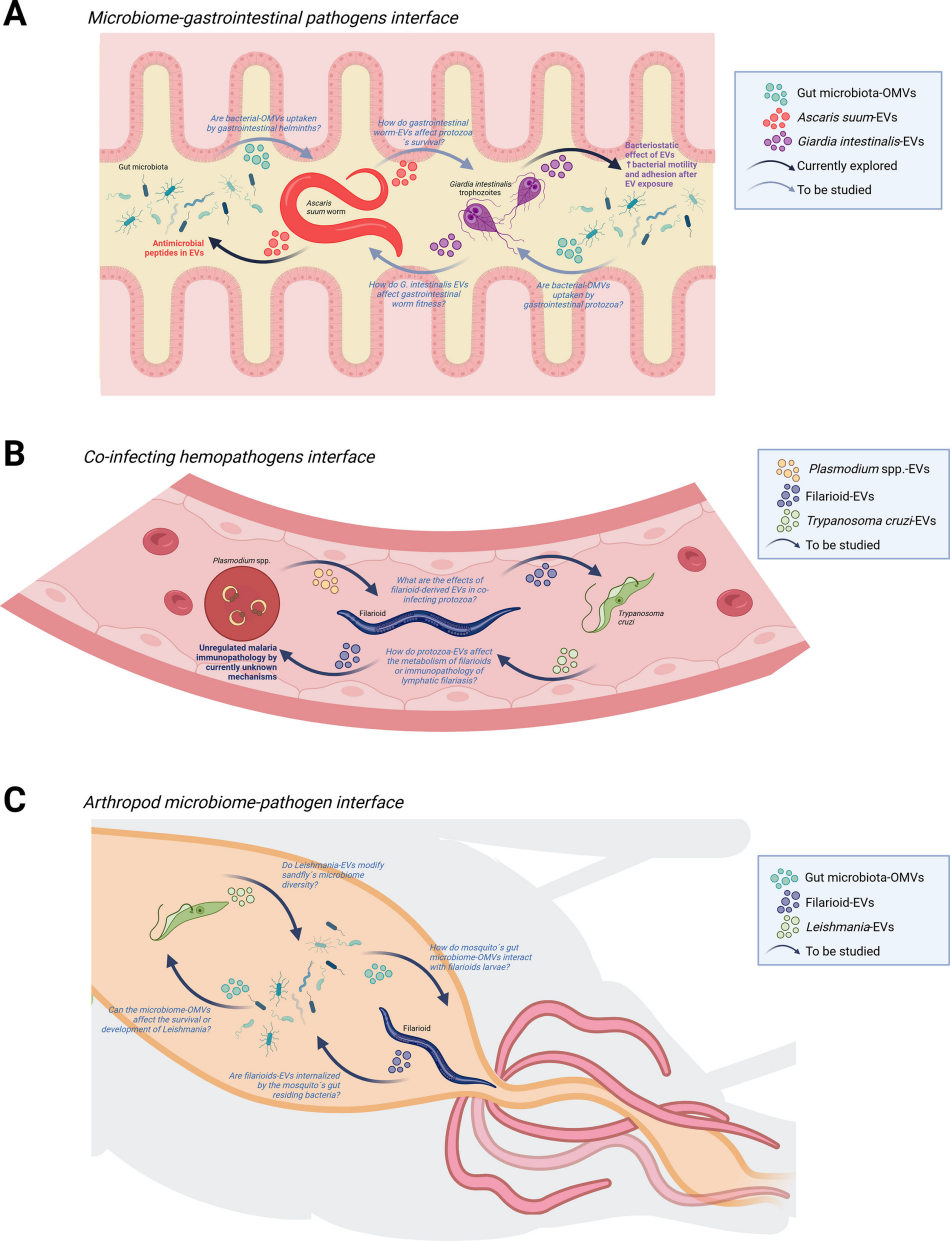
Key questions for the study of transmicrobe EV communications. (**A**) Proposed example studies in the microbiome-gastrointestinal pathogens interface, (**B**) coinfecting pathogens in the bloodstream, and (**C**) the interactions between arthropod-associated microbiome and protozoa or helminth pathogens. Outstanding questions are denoted in italic letters, and studied mechanisms are in bold letters.

A few analyses have addressed the cross talk between microorganisms. This is particularly relevant in the host’s gut microenvironment, where a high microbe diversity is found, or in blood-borne pathogens, where potential direct communication may occur. Transmicrobe interactions have focused on the vector-borne pathogen or helminth-microbiome interfaces. For instance, it is well understood that the gut microbiome’s alpha diversity in people with gastrointestinal helminths is increased, accompanied by significant bacterial taxonomic reassortment (45). Moreover, helminth-gut microbiome cross talk via EVs has been explored with antimicrobial peptides described in the excretion-secretion products and EVs of the roundworm *Ascaris suum* (46–48). Additionally, *Giardia intestinalis* EVs have shown a bacteriostatic effect over *Enterobacter cloacae* and *Enterobacter faecalis* and can increase the motility and adhesion of *Escherichia coli* HB101 to intestine cells, most likely via EV-derived RNA (49). Although the internalization of helminth or protozoa EVs by bacteria, protozoa, or fungi, and vice versa, has not been demonstrated, the ways in which helminth or protozoa EVs or ESPs affect the host’s microbial communities have potential applications in the design of alternative treatments against gastrointestinal pathogens (50) (Fig. 1A). On the other hand, gaining knowledge about the immunomodulatory properties of helminth-bacterial EV interactions could be useful in combating STHs, which affect approximately 1.5 billion people worldwide (51) and other gastrointestinal helminths common in pets and livestock.

Interactions between blood-borne pathogens, such as protozoa, bacteria, fungi, or helminths, may lead to direct EV exchange between microbes with consequences in their development, host immunoregulation, and pathogenesis. Animal models of concomitant LF and non-cerebral malaria have shown that clinical manifestations of malaria worsen when mice are amicrofilaremic, suggesting that these worms lead to an unregulated malaria immunopathology (52). LF pathogenesis is highly complex and depends, among other factors, on the presence of adult worms in lymphatic vessels and the circulation of microfilariae (53). Therefore, studying EV interactions between microfilaria and adult stages and malaria-infected RBCs could reveal new aspects for managing these infections (Fig. 1B).

EV transmicrobe cross talk plays a crucial role in the vector-pathogen interface, where intimate contact between sandflies, mosquitoes, or ticks occurs as bacteria, protozoa, or helminths replicate during their development (54). For example, it has been demonstrated that *Leishmania major* and *Leishmania infantum* actively produce EVs in the midgut of their sandfly vector, which are later co-egested during their blood meal (55). In addition, infection of *Ixodes scapularis* ticks with the bacteria *Anaplasma phagocytophilum* modifies tick-EV morphology and increases the presence of carbonylated proteins in tick EVs when compared with controls (8). Moreover, mice injected with *Dermacentor andersoni* EVs and *Francisella tularensis* showed decreased splenomegaly and lower levels of inflammatory cytokines in the animals (8). However, as with helminths, it is unknown whether this interaction is mediated by the internalization of bacteria EVs into tick cells. In addition, vector arthropods carry their own microbiota, which alters their metabolism and immune responses to human pathogens (56). Some bacterial genera abundant in the gut microflora of *Triatoma dimidiata*, the vector of Chagas disease*,* have shown inhibitory effects on the growth of *Trypanosoma cruzi* (57). Therefore, direct interactions via EVs between vector-associated microflora and *T. cruzi* should be tested, since this may render potential nonchemical compounds that reduce *T. dimidiata* competence as a vector. This strategy could also be applied to other vector-borne pathogens, *Plasmodium* spp. in *Anopheles* mosquitoes, *Leishmania* spp. in sandflies, or *W. bancrofti* in *Aedes* and *Culex* mosquitoes (Fig. 1C).

## CONCLUSIONS

Studying transmicrobe interactions via EVs will provide a broader perspective on the immunomodulatory pathways followed during infections, since this may reflect the reality observed during coinfections. Most importantly, it will offer novel tools for controlling pathogenic agents in both vertebrate and invertebrate hosts. Therefore, direct efforts should focus on elucidating EV uptake between the microorganisms from different kingdoms, understanding the molecular and metabolic effects of this cross talk, and identifying candidates that could later be used to influence the metabolism of target pathogens. This may require experimental infections using spheroids, organoids, and animal models and may be complemented with transkingdom network analysis as a tool to assess the impact of different factors that modify the host’s microbiota (58) or in this case transmicrobe communities.
